# The efficacy of bisphosphonates for osteoporosis in young Cushing’s disease patients with biochemical remission: a retrospective cohort study

**DOI:** 10.3389/fendo.2024.1412046

**Published:** 2024-06-21

**Authors:** Quanya Sun, Wanwan Sun, Hongying Ye, Shuo Zhang

**Affiliations:** Department of Endocrinology and Metabolism, Huashan Hospital, Fudan University, Shanghai, China

**Keywords:** Cushing’s disease, young patients, osteoporosis, bisphosphonates, bone turnover markers

## Abstract

**Background:**

Patients with Cushing’s disease (CD) often experience slow recovery of bone mineral density (BMD), and the effectiveness of anti-osteoporosis drugs in young CD patients who have achieved biochemical remission after surgery is not well understood. Therefore, we aimed to explore whether bisphosphonates could help accelerate the recovery of osteoporosis in young CD patients with remission.

**Methods:**

We retrospectively enrolled 34 young patients with CD who achieved postoperative biochemical remission. All patients suffered from osteoporosis before surgery and were divided into postoperative bisphosphonate treatment group (16 cases) and without bisphosphonate treatment group (18 cases). Clinical data, BMD (Z Value), and bone turnover markers were collected at the time of diagnosis and one year after successful tumor resection.

**Results:**

The Z values in the lumbar spine showed slight improvement in both groups at follow-up compared to baseline, but this improvement was not statistically significant. There was no significant difference observed between the two groups at follow-up. One year after operation, bone formation markers (OC and P1NP) were significantly higher than those at baseline in both groups. However, OC and P1NP in the bisphosphonate treatment group were lower than those in control group at one year follow-up. In without bisphosphonate treatment group, β-CTX from follow-up visit was higher than that at baseline, while no significant difference was observed in the bisphosphonate treatment group before and after surgery.

**Conclusion:**

Young patients with Cushing’s disease combined with osteoporosis might not benefit from bisphosphonate therapy for osteoporosis recovery in the first year after achieving biochemical remission.

## Introduction

Osteoporosis is one of common complications of Cushing’s syndrome (CS). 40–78% of CS patients have osteopenia at diagnosis and 22–57% have osteoporosis (1). Previous studies reported non-violent fractures in 16–50% of patients with CS at diagnosis (1–5).

The pathophysiological mechanism of glucocorticoid (GC)-induced osteoporosis is very complex. The main feature is a persistent decrease in bone formation accompanied by an early transient increase in bone resorption, which directly acts on osteoblasts, osteoclasts, and osteocytes (6–9). In addition, GC also can lead to bone loss through indirect effects, mainly including decreased sex hormone levels, intestinal and renal calcium absorption and reabsorption, muscle mass and mechanical sensitivity and increased parathyroid hormone levels, etc. (10).

Prevention strategies for osteoporosis in patients treated with long-term exogenous hormones were relatively mature, and drugs promoting bone formation or inhibiting bone resorption should be used. However, osteoporosis was often ignored in patients with Cushing’s syndrome. Previous studies had shown that BMD of patients with CS improved after achieving biochemical remission (11), but some patients still had osteoporosis for several years after remission, even though their BMD were improved compared to preoperative levels (1). A study showed that BMD increased due to high turnover of bone after CS remission, and no additional anti-osteoporotic treatment was considered (12). However, till now it remained unclear whether anti-osteoporosis treatment could help accelerate the recovery of osteoporosis in young CD patients with biochemical remission after surgery.

Therefore, the aim of this study was to determine the efficacy of bisphosphonates for osteoporosis in young Cushing’s disease (CD) patients with biochemical remission.

## Materials and methods

### Subjects

**Figure 1:**
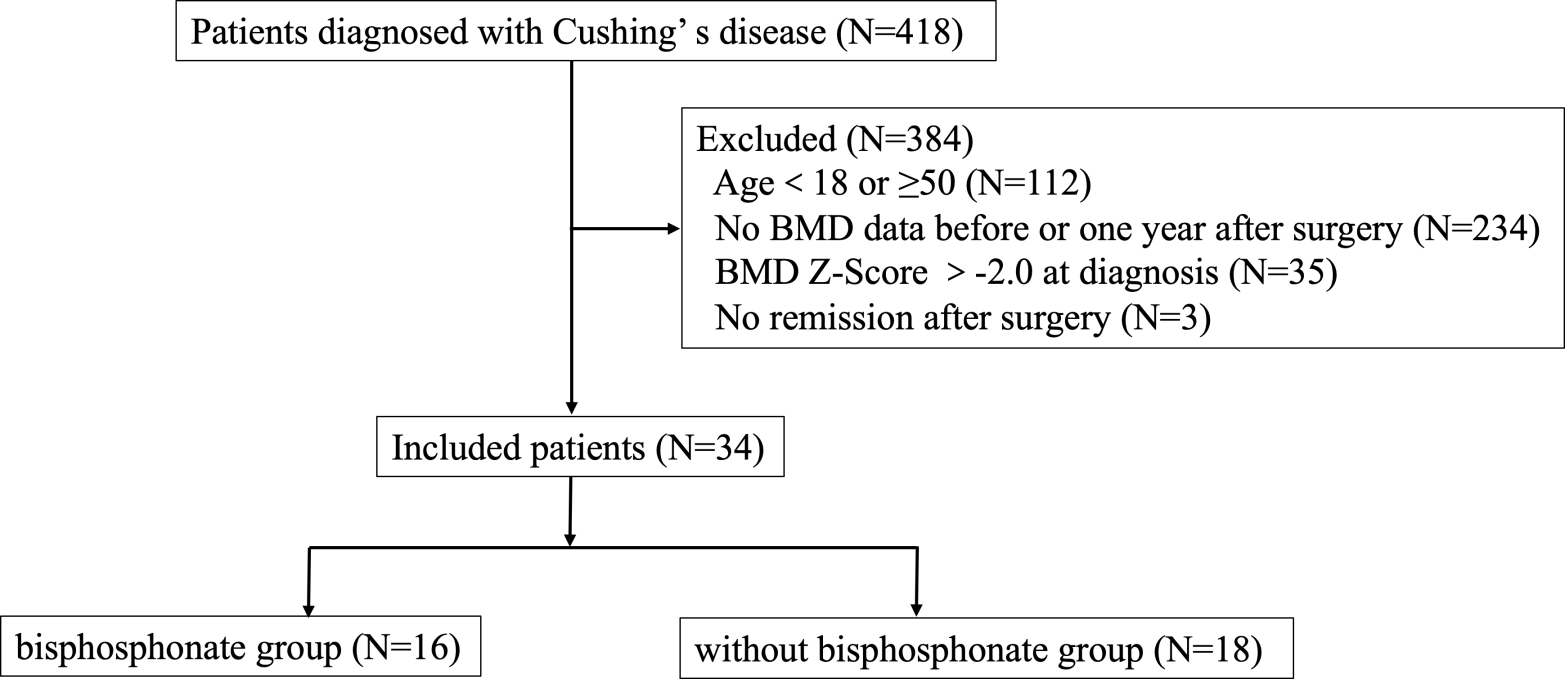
Research flow chart.

This study was a retrospective cohort study and was approved by the Human Investigation Ethics Committee at Huashan Hospital (No.2017M011). Thirty-four young CD patients combined with osteoporosis at diagnosis who were hospitalized in the Department of Endocrinology, Huashan Hospital, Fudan University from January 2010 to February 2021 were included. Patients’ selection was shown in **Figure 1**.

Inclusion criteria were as follows: 1) the diagnostic criteria for Cushing’s disease were met, and the pituitary ACTH adenoma was confirmed by surgical pathology, 2) men ≥18 years old but younger than 50 years old at diagnosis; premenopausal women ≥ 18 years old and young women(<50 years old) with menstrual abnormalities which were associated with CD, 3) Z-score of BMD in lumbar spine or femoral neck ≤-2.0 at diagnosis of Cushing’s disease or with a history of fragility fractures, 4) attaining biochemical remission after transsphenoidal surgery, 5) receiving regular follow-up and bone mineral density was measured in our hospital at diagnosis and one year follow-up.

Enrolled patients were divided into two groups based on whether using bisphosphonates treatment after surgery or not. Biochemical remission of Cushing’s disease was defined as morning serum cortisol <2μg/dL (<55nmol/L) within the week after surgery and although serum cortisol at 8:00 a.m. was≥2 µg/dl or back to normal range immediate after surgery, it became hypocortisolemic at subsequent evaluation(s) and without relapse during the follow-up (13–15). Meanwhile, relapse was excluded by cortisol value < 1.8 µg/dL after 1-mg dexamethasone suppression test (DST) and 24-hour urinary free cortisol (UFC) in normal range (13).

Exclusion criteria included: 1) having comorbidities affecting BMD (e.g., hyperparathyroidism, hyperthyroidism, primary hypogonadism, rheumatic immune disease, gastric bypass, inflammatory bowel disease, etc.), 2) long-term use of glucocorticoid drugs for the treatment of immune related diseases (except for hypopituitarism hormone replacement therapy) or other drugs that significantly affect bone metabolism, 3) use of anti-osteoporosis drugs before surgery, 4) postoperative treatment with anti-osteoporotic drugs other than bisphosphonate, 5) Cushing’s syndrome other than pituitary origin, 6) loss of follow up, 7) uncured or relapse of CD during the follow up.

### Clinical and biochemical methods

We collected data on demographic characteristics, duration of CD-related signs and symptoms, comorbidities, medications, laboratory tests, and bone mineral density.

Endocrine hormones included cortisol (F), 24-hour urinary free cortisol (24hUFC), adrenocorticotropic hormone (ACTH); growth hormone (GH), insulin-like growth factor (IGF-1), prolactin (PRL), luteinizing hormone (LH), follicle stimulating hormone (FSH), estrogen (E2), progesterone (P), testosterone (T), thyroid stimulating hormone (TSH), and free thyroxine (FT4). Hormonal measurements were carried out by chemiluminescence assay (Advia Centaur CP). Bone metabolism markers included osteocalcin (OC), type I procollagen amino-terminal peptide (P1NP), type I collagen C-terminal peptide degradation product (CTX), parathyroid hormone (PTH), 25-hydroxyvitamin D [25(OH)VD], and they were measured in a Roche Cobas e411 analyzer using immunometric assays (Roche Diagnostics, Indianapolis, IN, USA).

Bone mineral density was measured by dual-energy X-ray absorptiometry of American HOLOGIC company Discovery type W in all patients at diagnosis of CD and one year follow-up after surgery. Z value was used for young CD patients and Z value = (measured value - mean bone mineral density of peers)/standard deviation of BMD of peers. In this study, osteoporosis was defined as a Z-value of -2.0 or lower or with a history of fragility fractures.

All patients were administered with 20mg of hydrocortisone 3 times daily after surgery to avoid steroid withdrawal syndrome, with a 10-day taper afterward. When hydrocortisone was reduced to 10mg once a day for 10 days, the patient was followed up for the first time after surgery. Then the hormone replacement dose was adjusted based on the patient’s blood level obtained before that day’s glucocorticoid intake and urine cortisol level. All patients were administered with calcium carbonate D3 tablet (one tablet a day, consisting of calcium 600mg and D3 125U) and vitamin D (0.25ug a day) at diagnosis of osteoporosis till the last follow-up.

### Statistical analyses

Normal distributed continuous variables were expressed as mean values ± standard deviation (s.d.). Median, 25th percentile, and 75th percentile (Median [P25, P75]) for variables without a normal distribution. Independent t-tests for normally distributed continuous variables and non-parametric tests for variables without a normal distribution were used to compare data between groups. SPSS 20.0 (SPSS) was used. A two-tailed P value <0.05 was considered statistically significant.

## Results

### Patients’ characteristics at baseline

418 CD patients were hospitalized in the Department of Endocrinology, Huashan Hospital from January 2010 to February 2021. A total of 34 patients were included in our study, with an average age of 33.06 ± 7.37 years, 13 males (38.24%) and 21 females (61.76%). Sixteen patients were treated with bisphosphonates postoperatively (bisphosphonate group, including zoledronic acid and alendronate sodium), and eighteen patients were not treated with bisphosphonates postoperatively (without bisphosphonate group). Characteristics of the two groups were summarized in **Table 1**. Although there was a significant different in disease duration, there were no differences in age, gender, BMI, the proportion of hypertension, diabetes, dyslipidemia, liver function, kidney function, serum calcium, PTH, vitamin D level, bone metabolism markers, cortisol level, thyroid function, and growth hormone level between the two groups at baseline. Meanwhile, there was no significant difference in Z score of lumbar vertebra and femoral neck between two groups, -2.49 ± 0.56 (CV%=22.49%) vs-2.85 ± 0.61 (CV%=21.40%) and -1.74 ± 0.78 (CV%=44.83%) vs -1.93 ± 0.80 (CV%=41.45%) respectively. Therefore, the impact of different disease duration on the results was relatively small.

**Table 1:** Clinical Baseline Characteristics of Patients in two groups.

	Without Bisphosphonate	With Bisphosphonate	P value
N, %	18, 52.94%	16, 47.06%	/
Age (years)	33.11 ± 7.82	33.00 ± 7.09	0.9658
Male N, %	5, 27.78%	8, 50%	0.1832
Cushing duration(years)	5.51 ± 5.97	1.72 ± 1.01	0.0179
Fracture N, %	6/18, 33.3%	2/16, 12.5%	0.233
BMI (kg/m2)	26.32 ± 4.06	24.62 ± 2.93	0.1994
HBP N, %	14, 93.33%	14, 87.5%	0.5853
DM N, %	6, 54.55%	7, 87.5%	0.1271
Dyslipidemia N, %	10, 62.5%	6, 46.15%	0.3787
ALT (U/L)	27.50 (22.00–56.50)	39.00 (25.00–44.00)	0.7509
AST (U/L)	19.50 (17.00–27.00)	19.00 (15.00–22.00)	0.9376
Scr (mmol/L)	67.06 ± 22.33	68.31 ± 16.39	0.8682
24hUFC	318.38 (210.48–763.40)	473.83 (277.20–609.62)	0.7593
F 8am (μg/dL)	29.74 (23.62–35.91)	28.15 (25.57–35.18)	0.8566
F24 pm (μg/dL)	23.26 (16.35–33.31)	23.05 (20.51–27.78)	0.7486
TSH (mIU/L)	1.40 (0.73–1.57)	0.63 (0.52–1.01)	0.0124
FT3(pmol/L)	3.54 (3.28–4.01)	3.45 (3.30–3.91)	0.7299
FT4 (pmol/L)	13.10 (11.70–15.75)	13.20 (10.93–14.03)	0.3488
IGF1 Index	0.59 (0.48–0.68)	0.71 (0.47–0.86)	0.5211
25(OH)VD (nmol/L)	45.55 (37.13–62.10)	43.64 (40.10–51.80)	0.2432
Serum calcium	2.30 (2.25–2.37)	2.29 (2.25–2.37)	0.828
PTH (pg/ml)	36.30 (27.40–59.80)	56.60 (35.40–63.10)	0.1821
OC (ng/ml)	5.90 (2.40–8.03)	6.80 (4.50–8.60)	0.6562
P1NP (ng/ml)	34.72 (23.22–41.79)	22.57 (15.93–30.53)	0.3182
β-CTX(ng/ml)	0.42 (0.16–0.66)	0.59 (0.27–0.90)	0.526
Z score of lumbar vertebra	-2.49 ± 0.56	-2.85 ± 0.61	0.0811
Z score of femoral neck	-1.74 ± 0.78	-1.93 ± 0.80	0.4962

Normal range: ALT:7–40 U/L,AST: 13–35 U/L,Scr41–73 mmol/L, 24hUFC:30.15–129.13ug/24h,F 8am6.2–19.4ug/dlL, TSH0.27–4.2mIU/L, FT3: 3.1–6.8 pmol/L,FT4:12–22 pmol/L,IGF1 Index:<1, 25(OH)VD:>50 nmol/L, Serum calcium: 2.11–2.52, PTH 15–65 pg/ml,OC 4.11–21.87 ng/ml,P1NP:8.53–64.32 ng/ml, β-CTX:0.07–0.68 ng/ml.

### One year after achieving biochemical remission, BMD improved in both groups; however there was no significant difference between the two groups

For these patients with osteoporosis secondary to Cushing’s disease, the most important work was to remove the cause. Patients with a history of fragility fractures didn’t receive bisphosphonate after surgery partly because they refused to use it. As shown in **Table 2**, **Figures 2A, B**, there were no significant differences in the Z Score of lumbar vertebra and femoral neck between the two groups at baseline. The Z values in lumbar spine at one year follow-up of both groups were slightly improved but not significantly compared to baseline respectively. There was no significant difference in the Z score of lumbar vertebra [-2.40 ± 0.617 (CV%=25.71%) vs -2.81 ± 0.771 (CV%=27.44%), p=0.0766] or femoral neck [-1.9 ± 0.715 (CV%=37.63%) vs -2.01 ± 0.726 (CV%=36.12%), p=0.6378] between two groups at one year follow-up.

**Table 2:** Changes in bone mineral density and bone turnover markers before and 1 year after remission in the two groups.

	Without Bisphosphonate		P value	With Bisphosphonate		P value	P value#
	Baseline	1 year after remission		Baseline	1 year after remission		
Z score of lumbar vertebra	-2.49 ± 0.56	-2.40 ± 0.617	0.9638	-2.85 ± 0.61	-2.81 ± 0.771	0.1174	0.0766
Z score of femoral neck	-1.74 ± 0.78	-1.9 ± 0.715	0.3615	-1.93 ± 0.80	-2.01 ± 0.726	1.0000	0.6378
OC (ng/ml)	5.90 (2.40–8.03)	46.7(23.25–83)	0.000	6.80 (4.50–8.60)	33.8(14.46–49.27)	0.014	0.0381
P1NP (ng/ml)	34.72 (23.22–41.79)	353.5(124.9–501.2)	0.004	22.57 (15.93–30.53)	181.1(65.46–228.75)	0.002	0.0484
β-CTX(ng/ml)	0.42 (0.16–0.66)	0.97 (0.83–1.57)	0.006	0.59 (0.27–0.90)	0.72(0.47–1.50)	0.115	0.409
25(OH)VD (nmol/L)	45.55 (37.13–62.10)	61(49.2–84.1)	0.242	43.64 (40.10–51.80)	43.51(36.6–61)	0.569	0.0271
Serum calcium	2.30 (2.25–2.37)	2.28 (2.21–2.35)	0.427	2.29 (2.25–2.37)	2.23 (2.19–2.38)	0.164	0.664
PTH (pg/ml)	36.30 (27.40–59.80)	25.95(19.05–40.95)	0.311	56.60 (35.40–63.10)	33.6(21.7–50.2)	0.014	0.72

#Data from without Bisphosphonate group 1 year after remission compared with bisphosphonate group 1 year after remission.

**Figure 2:**
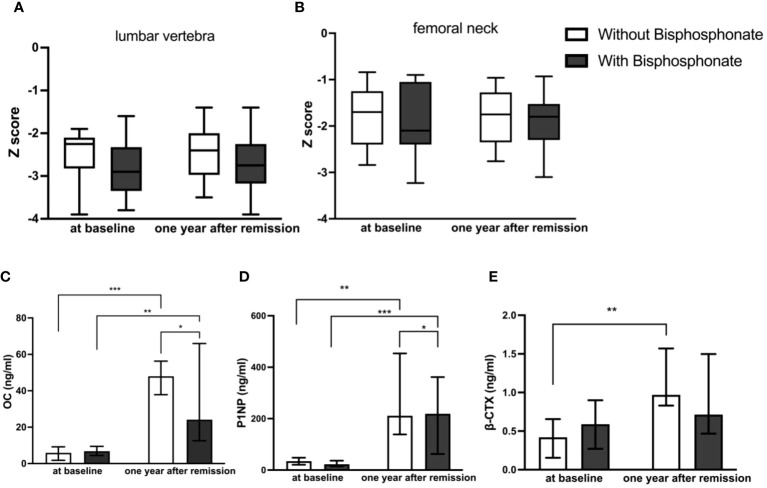
Comparison of BMD and bone turnover markers at baseline and one year after remission between bisphosphonate-treated and non-bisphosphonate-treated groups. **(A)** Z Score of lumbar vertebra; **(B)** Z Score of femoral neck; **(C)** levels of OC; **(D)** levels of P1NP; **(E)** levels of β-CTX. *P < 0.05, **P < 0.01, ***P < 0.001.

### At one year follow-up, bone formation markers increased obviously in both groups compared to those at diagnosis, and they increased higher without bisphosphonate treatment

As shown in **Table 2**, **Figures 2C–E**, there were no significant differences in bone turnover markers including OC, P1NP, and β-CTX between the two groups at baseline. Serum OC levels were significantly higher than those before surgery in both groups at one year follow-up after achieving remission respectively (5.90 (2.40–8.03) ng/ml vs 46.7 (23.25–83) ng/ml in control group, p<0.0001, and 6.80 (4.50–8.60) ng/ml vs 33.8 (14.46–49.27) ng/ml in treatment group, p=0.009). However, the serum OC level in the control group at follow-up was significantly higher than that in the treatment group [46.7 (23.25–83) ng/ml vs 33.8 (14.46–49.27) ng/ml, p=0.0381]. Serum P1NP levels were also significantly higher than those before surgery in both groups at follow-up after achieving remission of Cushing’s disease respectively (34.72 (23.22–41.79) ng/ml vs 353.5(124.9–501.2) ng/ml in control group, p=0.003, and 22.57 (15.93–30.53) ng/ml vs 181.1(65.46–228.75) ng/ml in treatment group, p=0.001). Similarly, the serum P1NP level at follow-up in the control group was significantly higher than that in the treatment group [353.5 (124.9–501.2) vs 181.1 (65.46–228.75) ng/ml, p=0.0484].

In the group without bisphosphonate treatment, β-CTX at one year after remission was higher than that before surgery [0.97 (0.83–1.57) vs 0.42 (0.16–0.66) ng/ml, p=0.006]. However, there was no significant difference in the bisphosphonate treatment group between baseline and follow-up [0.59 (0.27–0.90) vs 0.72(0.47–1.50) ng/ml, p=0.115]. No significant difference was seen for β-CTX level at follow-up between the two groups [0.97 (0.83–1.57) vs 0.72(0.47–1.50) ng/ml, p=0.409].

## Discussion

Osteoporosis is one of common complications of Cushing’s disease and the recovery of bone mineral density after remission is a slow process. An important clinically question is whether young patients with CD after remission would benefit from anti-osteoporotic drugs such as bisphosphonates. To our knowledge, this study was the first well-powered retrospective cohort study of the efficacy of bisphosphonates for osteoporosis in young CD patients with biochemical remission. Our data showed that BMD improved slowly in young CD patients with remission at the first- year follow-up regardless of whether bisphosphate was used or not, and no significant difference in BMD improvement was observed between two groups at follow-up.

It was well known that after cure of Cushing’s syndrome, there was a long recovery period for BMD. It had been shown that full recovery from BMD in cured adult CS patients could take up to a decade or more (1, 16). However, Hermus (17) had shown that some patients had a 2% or more reduction in BMD in the short term after surgery, especially in the first 6 months after surgery, and did not show consistent BMD increases until 24 months after surgery. It also showed that there was a highly significant inverse correlation between age and increase of BMD in the lumbar spine after surgery (17). The lack of significant improvements in BMD in our results might be related to the short duration of follow-up.

Current studies of endogenous Cushing’s syndrome had shown that bone metabolism was characterized by decreased bone formation and increased bone resorption, consistent with the classical effects of glucocorticoids (11). Successful treatment of endogenous Cushing’s syndrome resulted in a strong activation of bone turnover, characterized by increased bone formation and resorption. A retrospective study by Pepijn van Houten showed that sustained improvement in BMD continued for up to 20 years after CD treatment, and a large proportion of patients in this cohort were treated with anti-osteoporotic drugs (1). The study also showed that patients not receiving anti-osteoporosis drugs experienced significant spontaneous improvement in mean BMD. However, this retrospective study could not be used to answer the clinical question of whether anti-osteoporotic therapy was beneficial due to selection bias in enrolled patients. Leah T Braun showed that within 2 years of successful surgical remission in patients with Cushing’s syndrome, markers of bone formation suggested a high rate of bone turnover, resulting in a significant net increase in BMD in the majority of patients. The results strongly suggested that an observational approach to bone phenotype was justified as long as CS remission was assured (12). However, this retrospective study mentioned a significant mismatch in baseline BMD between the two groups (anti-osteoporotic medication group and without anti-osteoporotic medication group) and did not describe the type of anti-osteoporosis drugs (promoting bone formation or inhibiting bone resorption or both). Somma’s prospective study showed that a significant increase in lumbar and femoral BMD was observed in 21 CD patients who achieved remission after surgery and were either treated with alendronate for 12 months or not (18). It should be noted that this study included postmenopausal women, and there were no direct comparisons of clinical data, bone mineral density, and bone turnover markers at baseline and follow-up between the two groups.

Our study also showed that even bone formation markers increased at follow-up in bisphosphonate group, they were significantly lower compared to non- bisphosphonate users. Since bone metabolism was in a state of high turnover in the initial stage of biochemical remission from Cushing’s disease, our results indicated that bisphosphonates might affect bone formation in the first year after remission and was not conductive to the improvement of BMD. The mechanism of bisphosphonates in the treatment of osteoporosis lied in their high affinity with skeletal hydroxyapatite, allowing them to specifically bind to actively remodeling bone surface and inhibit the function of osteoclasts, thereby inhibiting bone resorption. Studies had shown that while bisphosphonates strongly inhibited bone resorption, they also significantly reduced bone formation. This reduced formation was often attributed to mechanisms that maintained the resorption/formation balance during remodeling (19).

There are evidence-based guidelines available for assessing fracture risk during long-term exogenous glucocorticoid(GC) therapy in adults, as well as for initiating and selecting anti-osteoporosis therapy. Specifically, for patients at risk of fracture taking GC ≥2.5 mg/day for >3 months, treatment options include bisphosphonates, denosumab, or PTH analogs. Although there is currently no definitive evidence-based treatment regarding the choice and efficacy of anti-osteoporosis after glucocorticoid withdrawal, it is widely accepted that treatment should be continued based on bone density and fracture risk assessment. For patients at a high fracture risk level (T≤-2.5), it is recommended to either continue their current anti-osteoporosis treatment or switch to an alternative medication. The main challenge faced by individuals with endogenous glucocorticoid induced osteoporosis (GIOP) is that exogenous GIOP is not exactly the same as endogenous GIOP. Therefore, it is not appropriate to apply the same strategies of exogenous GIOP for CD patients with remission. The findings of this study indicated that bisphosphonate therapy might not be beneficial for osteoporosis recovery in CD patients achieving biochemical remission (20).

Our study, limited by retrospective clinical studies, a small sample size, and a short follow-up duration, might not optimally answer the question of whether patients with CD achieving remission would benefit from bisphosphonate therapy, although it was a relatively well-designed retrospective cohort study and reached the maximum number after strict inclusion criteria and matching baseline characteristics as much as possible. Therefore, prospective randomized controlled clinical trials with longer duration were needed in the future.

In conclusion, our study suggested that young patients with Cushing’s disease combined with osteoporosis might not benefit from bisphosphonate therapy for osteoporosis recovery in the first year after achieving biochemical remission.

## Data availability statement

The raw data supporting the conclusions of this article will be made available by the authors, without undue reservation.

## Ethics statement

The studies involving humans were approved by the Human Investigation Ethics Committee at Huashan Hospital. The studies were conducted in accordance with the local legislation and institutional requirements. The participants provided their written informed consent to participate in this study.

## Author contributions

QS: Writing – original draft. WS: Writing – original draft, Formal analysis, Data curation. HY: Writing – review & editing, Supervision, Resources. SZ: Writing – review & editing, Supervision.
